# Pharmacological functions of salidroside in renal diseases: facts and perspectives

**DOI:** 10.3389/fphar.2023.1309598

**Published:** 2024-01-08

**Authors:** Qiong Liu, Jianzhu Chen, Anqi Zeng, Linjiang Song

**Affiliations:** ^1^ School of Medical and Life Sciences, Chengdu University of Traditional Chinese Medicine, Chengdu, Sichuan, China; ^2^ Translational Chinese Medicine Key Laboratory of Sichuan Province, Sichuan Academy of Chinese Medicine Sciences, Sichuan Institute for Translational Chinese Medicine, Chengdu, Sichuan, China

**Keywords:** salidroside, diabetic nephropathy, renal interstitial fibrosis, acute kidney injury, inflammation, renal cell carcinoma

## Abstract

*Rhodiola rosea* is a valuable functional medicinal plant widely utilized in China and other Asian countries for its anti-fatigue, anti-aging, and altitude sickness prevention properties. Salidroside, a most active constituent derived from *Rhodiola rosea*, exhibits potent antioxidative, hypoxia-resistant, anti-inflammatory, anticancer, and anti-aging effects that have garnered significant attention. The appreciation of the pharmacological role of salidroside has burgeoned over the last decade, making it a beneficial option for the prevention and treatment of multiple diseases, including atherosclerosis, Alzheimer’s disease, Parkinson’s disease, cardiovascular disease, and more. With its anti-aging and renoprotective effects, in parallel with the inhibition of oxidative stress and inflammation, salidroside holds promise as a potential therapeutic agent for kidney damage. This article provides an overview of the microinflammatory state in kidney disease and discuss the current therapeutic strategies, with a particular focus on highlighting the recent advancements in utilizing salidroside for renal disease. The potential mechanisms of action of salidroside are primarily associated with the regulation of gene and protein expression in glomerular endothelial cells, podocytes, renal tubule cells, renal mesangial cells and renal cell carcinoma cell, including TNF-α, TGF-β, IL-1β, IL-17A, IL-6, MCP-1, Bcl-2, VEGF, ECM protein, caspase-3, HIF-1α, BIM, as well as the modulation of AMPK/SIRT1, Nrf2/HO-1, Sirt1/PGC-1α, ROS/Src/Cav-1, Akt/GSK-3β, TXNIP-NLRP3, ERK1/2, TGF-β1/Smad2/3, PI3K/Akt, Wnt1/Wnt3a β-catenin, TLR4/NF-κB, MAPK, JAK2/STAT3, SIRT1/Nrf2 pathways. To the best of our knowledge, this review is the first to comprehensively cover the protective effects of salidroside on diverse renal diseases, and suggests that salidroside has great potential to be developed as a drug for the prevention and treatment of metabolic syndrome, cardiovascular and cerebrovascular diseases and renal complications.

## Introduction

Renal diseases pose a global public health issue. Based on research data from the American Society of Nephrology, the European Renal Association–European Dialysis and Transplant Association and the International Society of Nephropathy, at least 850 million people worldwide are currently suffering from renal disease (Jager et al., 2019). Reasons for renal disease are widely available, with the most common being diabetic nephropathy, hypertensive nephropathy, primary glomerulonephritis, obstructive nephropathy, and lupus nephritis, etc., (Carriazo et al., 2020; Gasparotto et al., 2020; Samsu, 2021; Wang et al., 2022). In the context of the vast population of chronic kidney disease patients today, delaying the progression of the disease is a major problem to be addressed. With the advancement of modern traditional Chinese medicine, the significant value of traditional Chinese medicine with hypoglycemic, anti-inflammatory, and renal protective properties in the field of medical disease treatment is increasingly being recognized (Zhao et al., 2020). Furthermore, through the utilization of various techniques to identify active compounds, uncover potential mechanisms of action, and meticulously design experiments, efforts are being made to validate the effectiveness and safety of their use in patients with diverse renal disorders.

*Rhodiola rosea* has been utilized in traditional Asian medical therapies for thousands of years, which is extensively used for the treatment of various ailments including cardiovascular diseases, kidney diseases, nervous system disorders, liver fibrosis, immune system disorders, and other medical conditions (Ishaque et al., 2012; Li et al., 2017; Pu et al., 2020; Jin et al., 2022). Modern chemical analysis has identified 104 compounds from Rhodiola rosea including the main components salidroside, pyridrde, rhodiosin, rosavidne (Panossian et al., 2010; Chen et al., 2021). 2-(4-Hydroxyphenyl) ethyl β-D-glucopyranoside (salidroside), the most effective active substance for the pharmacological action of *Rhodiola rosea*, is widely present in all species of Rhodiola L. As modern pharmacological studies delve deeper, researchers have unveiled that salidroside, the main monomer of *Rhodiola rosea*, has a variety of essential biological activities, which can anti-aging, boost immunity, better the cardiovascular system, defend the kidney, while suppressing the proliferation and invasion of many tumor cells (Li et al., 2009; Magani et al., 2020; Hu et al., 2021; Han et al., 2022). These findings not only indicate the diverse therapeutic potential of salidroside but also highlight its promising role in the development of novel pharmacological interventions for a wide range of health conditions.

In this review, we exhaustively summarize recent advances in the molecular mechanisms and therapeutic effects of salidroside for the treatment of renal diseases, thus deepening our understanding of its therapeutic potential and recommending it as a drug candidate for the administration of renal diseases (Figure 3; Table1). Such discoveries could have significant implications for the development of effective drugs in the field of renal disease management.

**TABLE 1:** Therapeutic potential and molecular mechanism of salidroside in animal or cellular models of renal disease.

Kidney disease	Model	Mechanism	Effect	References
Diabetic nephropathy	STZ-induced mice	SIRT1/PGC-1α axis	↓	Guo et al. (2018)
	STZ-induced rat	Akt/GSK-3β	↑	Ho and Shirakawa (2022)
	HG-induce rat and mesangial cell	TXNIP-NLRP3 inflammasome	↓	Ren et al. (2020)
	STZ-induced rat	TGF-β1; Wnt1/3a/β-catenin	↓	Liu and He (2019)
	STZ-induced rat	TGF-β1/Smad	↓	Qi et al. (2021)
	STZ-induced T1DM rats and HG-induced mesangial cell	AMPK/SIRT1	↑	Shati (2020)
	STZ-induced rats and proximal tubular epithelial cell	BIM	↓	Guo et al. (2018)
	HG-induced glomerular endothelial cell	ROS/Src/Cav-1	↓	Wu et al. (2016)
	HG-induced glomerular endothelial cell	HIF-1α	↑	Juhaszova et al. (2009)
	HG-induced mesangial cell	TGF-β; ERK1/2 phosphorylation	↓	Yin et al. (2009)
	HG-induced Podocyte	HO-1	↑	Lu et al. (2017)
Renal ischemia reperfusion injury	HK-2 in response to H/R	TLR4/NF-κB	↓	Sun et al. (2018)
	RIRI-rat and renal tubular epithelial cells	PI3K/AKT	↑	Tang et al. (2023)
	Embryonic kidney fibroblast	HIF-1α	↑	Zheng et al. (2012)
Acute kidney injury	LPS-induced mice	SIRT1/Nrf2	↑	Pan et al. (2023)
	A rat model of sepsis by cecal ligation and perforation	Inflammation; Apoptosis	↓	Fan et al. (2022)
Renal fibrosis	UUO- or folic acid -induced mice	TLR4/NF-κB and MAPK	↓	Li et al. (2019b)
	SAMP8 mice	Ferroptosis	↓	Yang et al. (2022b)
	ADR‐induced mice	β‐catenin	↓	Shati and Alfaifi (2020)
	ADR‐induced mouse	PI3K/Akt	↓	Liu and He (2019)
Renal cell carcinoma	Mouse xenograft model of RCC	JAK2/STAT3	↓	Lv et al. (2016)
Contrast-induced-nephropathy	Contrast-induced-nephropathy rat	Oxidative stress	↓	Xing et al. (2015)
Cobaltous chloride induce-hypoxia	Normal rat kidney tubular epithelia cells (NRK52E)	tubular epithelial-myofibroblast transdifferentiation	↓	Zhang et al. (2010)

## Overview of *Rhodisrola Rosea*

*Rhodiola rosea* belongs to the Sedum family and is endowed with abundant alpine medicinal plant resources. There are approximately 200 species of *Rhodiola rosea* worldwide, mostly distributed in the northern hemisphere with China as the distribution center of 3,500–5000 m altitude of limestone, granite mountain glacier, mountain beam grass or valley rocks, a few grow in the altitude of about 2000 m alpine grassland, forest scrub or ditch rocks near, such as North America, East Asia, Central Asia and Siberia and other regions (Zhang et al., 2014; Booker et al., 2016; Tao et al., 2019). *Rhodiola rosea* is 5–20 cm tall, with terminal cymes, flowering in July and August, pale yellow or purple red to red, stems and leaves bright green, often with fleshy, creeping rhizomes, typically growing in dense patches in unpolluted alpine areas (Fu et al., 2017). *Rhodiola rosea* is a traditional Chinese medicinal material, and its roots and stems can be utilized as medicine. Its chemical composition is relatively complex and abounds in secondary metabolites (Tolonen et al., 2003). The first use of *Rhodiola rosea* as a medicine can be traced back to 800AD in the “Four Medical Code” (rGyud‐bzhi in Tibetan, Si Bu Yi Dian in Chinese). “Shennong Materia Medica” listed *Rhodiola rosea* as the top medicine, using *Rhodiola rosea* has the effects of relaxing the body and nourishing blood, promoting blood circulation and stopping bleeding, clearing lung and relieving cough. It also appeared in the “Materia Medica” of many European countries, and its roots have been used as a “tonic herb” by folk herbalists in Northern Europe and Russia to fight fatigue, prevent infection, and treat wounds and tuberculosis (Tao et al., 2019). There is the cultivation of Rhodiola rosea plant in Europe and North America, often as a dietary supplement (Chiang et al., 2015). The use of its root was also spotted in the first Swedish National Pharmacopoeia (Panossian et al., 2010). At the beginning of the last century, Russian scientists classified it as adaptogen, which denotes the ability to enhance the body’s resistance to all sorts of physical and mental stresses, and is suitable for long-term consumption as an aid to the treatment of many diseases (Todorova et al., 2021). In the light of the “adaptogenic” effect similar to *Panax ginseng* and *Eleutherococcus senticosus*, it could be speculated that salidroside can bring high ecological economic and social benefits.

## General bioactivity of salidroside

**FIGURE 1:**
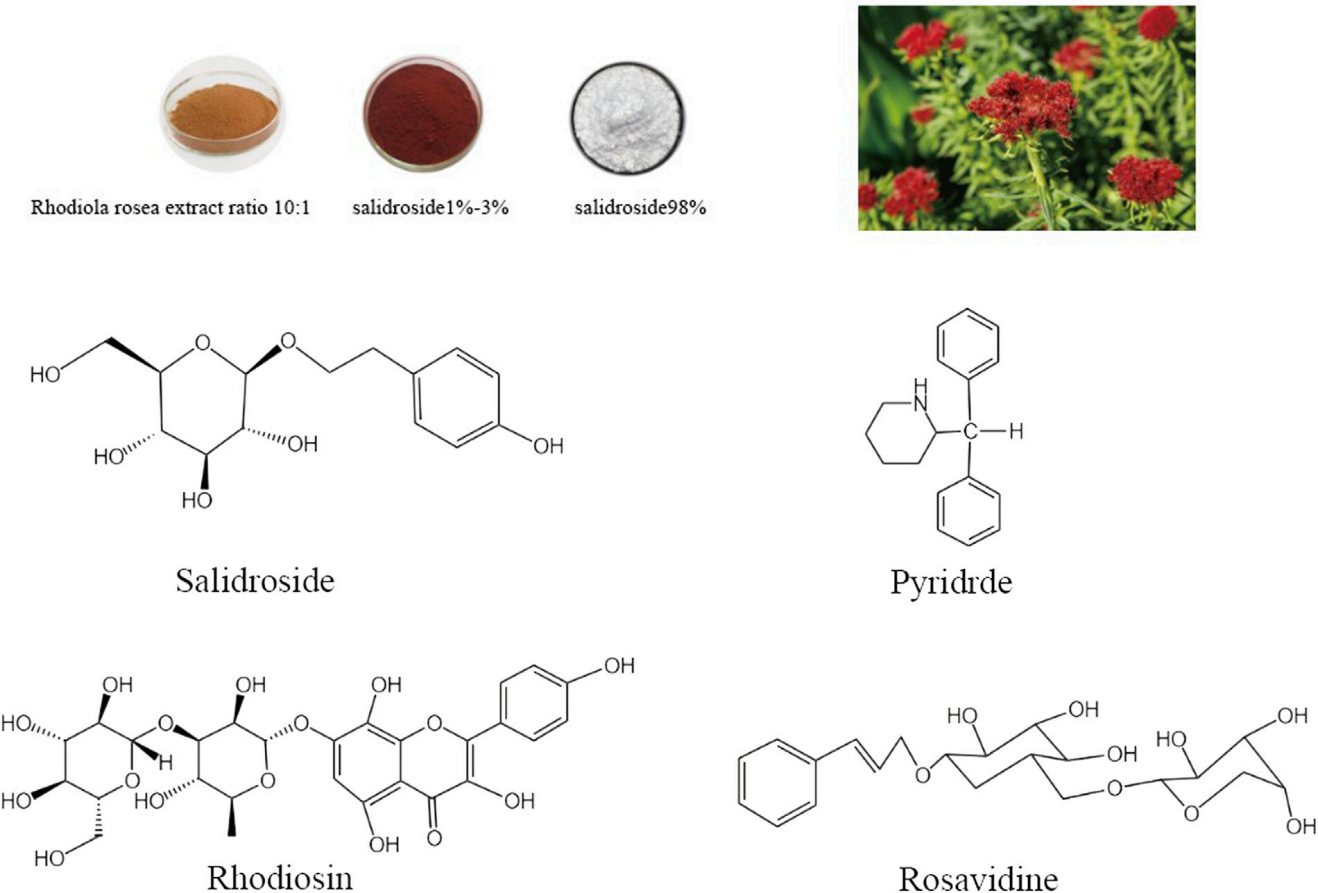
*Rhodiola rosea* and chemical structure formula of its main active components (Salidroside, Pyridrde, Rhodiosin, and Rosavidne).

**FIGURE 2:**
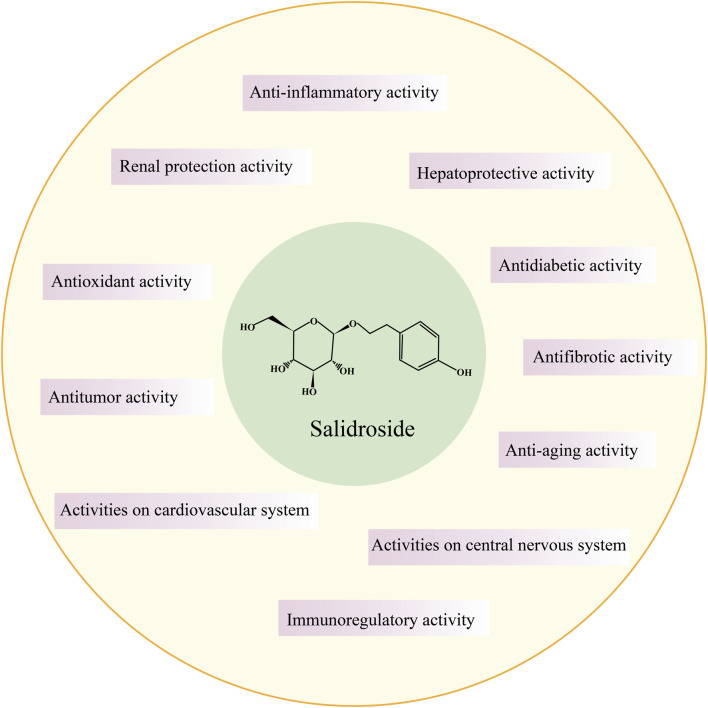
General pharmacological activity of salidroside.

In the 1960s, Soviet scholars evidenced that salidroside is the main active component of the adaptogenic plant *Rhodiola rosea*. Salidroside (molecular formula: C_14_H_20_O_7_, relative molecular mass: 300.3), is a phenylethanol compound with colorless and transparent needle-like crystals, which can be extracted from dried roots and rhizomes of *Rhodiola rosea* or dried whole herbs, or synthesized by biological, chemical, and biocatalytic routes (Figure 1) (Zhang et al., 2013; Fan et al., 2017). Salidroside is highly soluble in water, readily soluble in methanol and ethanol, and insoluble in ether. It can be decomposed into trimethylamine by the decomposition reaction of concentrated potassium hydroxide solution, which can be decomposed into one molecule of aglycone and glucose under the action of enzyme or acid. Pharmacokinetic experiments have shown that Salidroside is mainly metabolized by the liver and excreted by the kidney (Zhang et al., 2013). Previous studies have documented that Salidroside have versatile roles such as raising the immunity of the body, enhancing physical fitness, modernizing the hematopoietic function, anti-inflammation, anti-tumor, anti-hypoxia, hypoglycemia, anti-virus, anti-radiation, preventing plateau reaction, etc., (Figure 2) (Wang et al., 2009; Zhao et al., 2021; Wang Y. et al., 2023). Its unique adaptogen-like effect and two-way regulation can elevate the stability of the body to different stimuli and transform the abnormal indicators caused by varied etiologies to normal state.

Salidroside exhibits cytoprotective effects on human red blood cells against hydrogen peroxide-induced apoptosis, suggesting its potential as an adaptogen for enhancing human resilience to stress and fatigue (Qian et al., 2012). Salidroside inhibits excessive oxidative stress not only on red blood cells, but also inhibits oxidative stress in liver tissues of rats after intense exercise by scavenging free radicals and reactive oxygen species, improving exercise endurance and muscle glycogen and liver glycogen level, and alleviating exercise fatigue (Xu and Li, 2012). Salidroside can improve the immunity of the body and has the function of immune regulation. Compared with the oocyte group, salidroside combined with oocyte administration can promote the splenocyte proliferation induced by ovalbumin, generate a large amount of IL-2, IL-4, gamma interferon, IgG antibody, IgG1 antibody and IgG2b antibody, and increase the percentage of CD4^+^ and CD8^+^T cell subsets (Guan et al., 2011). Salidroside liposomes not only promote the maturation of dendritic cells *in vitro*, stimulate the proliferation of T cells and antigen presentation of DCs, but also regulate the balance of Th1 and Th2 pathways and stimulate immune response *in vivo*. Studies on old male rats have found that salidroside can fight immune aging and thus rejuvenate the body (Lu et al., 2013). Aging is an irreversible and inevitable process. Salidroside can delay aging by improving the activity of endogenous antioxidant enzymes, scavenging oxygen free radicals, inhibiting the secretion of inflammatory factors, promoting the secretion of growth hormone, repairing DNA damage, and enhancing immune response and cholinergic function. Many recent studies have confirmed the therapeutic value of salidroside in the treatment of age-related diseases, including Alzheimer, Parkinson, diabetes, cardiovascular disease, renal disease, etc (Zhang et al., 2020; Zhao et al., 2021; Yang et al., 2022a).

Collectively, there is no doubt that salidroside play a good inflammatory-immunomodulatory role in certain diseases, which can assist the body to restore homeostasis, build up the tolerance of body functions and protect various organ tissues as soon as possible under the stimulation of adverse environment. Therefore, salidroside have attracted attention as a potential compound for the treatment of renal diseases.

## Renal disease and microinflammation

Renal disease has become a significant global public health concern over the past few decade, and its burden continues to grow, with about 10% of adults worldwide affected by some form of chronic kidney disease (CKD) (Kalantar-Zadeh et al., 2021). It has been found that both CKD and acute kidney injury (AKI) patients have widespread microinflammation, which is an insidious and persistent immune state, different from the inflammatory response caused by microbial infections (Schindler, 2004; Mihai et al., 2018; Belo and Carvalho, 2023). A cohort study conducted in 2016 on 3,430 patients with chronic renal insufficiency revealed that decreased renal function was associated with elevated levels of serum inflammatory cytokines IL-6 and TNF-α (Amdur et al., 2016). Actually, microinflammation represents a significant yet easily overlooked aspect of renal disease.

Patients with CKD experience a decline in glomerular filtration rate (GFR), leading to the accumulation of toxins in the body, which further activates monocyte-macrophages to secrete inflammatory cytokines (Wu et al., 2020). Furthermore, the impaired renal function reduces the body’s ability to clear inflammatory factors, thereby maintaining a state of microinflammation (Poole et al., 1990). The presence of metabolic acidosis triggers an upregulation of angiotensin II(Ang II), aldosterone, and endothelin-1 levels, instigating inflammation and fibrosis (Wesso et al., 2020). Imbalance in the intestinal microbiota can disrupt the integrity of the intestinal barrier, which allow pathogenic bacteria and their harmful metabolites to cross the mucosal lining and enter the systemic circulation, incurring chronic inflammation and worsens renal injury (Li F. et al., 2019). Hyperglycemia can stimulate the production of AngII in mesangial cells and podocytes, resulting in heightened expression of inflammatory cytokines such as IL-6, TNF-α, and MCP-1 (Yoo et al., 2007). Accumulation of advanced glycation end-products (AGEs) promote the expression of inflammatory mediators (chemokines and cytokines) in damaged glomerular and tubular cells, which then induce extracellular matrix deposition as well as the differentiation and proliferation of myofibroblasts via NF-κB, JAK/STAT, and TGFβ/Smad signaling pathways (Sun et al., 2019; Steenbeke et al., 2022). In the setting of mitochondrial dysfunction and impaired autophagy, NLRP3 inflammasome, Toll-like receptors (TLR), and STING signal transduction pathway are activated in renal tubular cells and podocytes, mediating the occurrence of inflammation in AKI and CKD (Kimura et al., 2017; Chung et al., 2019; Jin et al., 2021; Bhatia and Choi, 2023). The downstream of AGEs pathway, PKC pathway, and polyol pathway exhibit oxidative stress, leading to glomerular endothelial cell injury, accompanied by the expression of molecules related to inflammatory pathways (Ma X. et al., 2023). Dyslipidemia triggers an inflammatory response in CKD, with excessive lipid deposition in the basement membrane, renal tubules and interstitium, stimulating macrophages to produce growth factors, cytokines, and mediators of mesangial cell proliferation as well as foam cell generation, contributing to arteriosclerosis and subsequent glomerulosclerosis and tubulointerstitial injury (Mitrofanova et al., 2023). The emergence of microinflammation in turn induce oxidative stress and worsen the disruption of glucose and lipid metabolism, rather than being merely inconsequential secondary harm (Rapa et al., 2019; Mitrofanova et al., 2023). Exposure of blood to bioincompatible dialysis membranes during renal replacement therapy in patients with end-stage renal disease can lead to the activation of monocytes and subsequent release of inflammatory factors into peripheral circulation (Olivier et al., 2020).

In brief, the microinflammatory state, intricately intertwined with renal diseases, is now recognized to be associated with impaired clearance of inflammatory cytokines, acid-base disturbance, volume overload, mitochondrial dysfunction, oxidative stress, lipid metabolic disorder, dysbiosis in intestinal microbiota and hyperglycemia (Olivier et al., 2020; Kadatane et al., 2023). Furthermore, the presence of microinflammation in patients with CKD also serves as a robust prognostic indicator, closely associated with various complications including malnutrition, anemia and adverse cardiovascular events (L et al., 2021). Undoubtedly, Improved management of microinflammation and oxidative stress is pivotal for decelerating the progression of CKD, ameliorating the prognosis, as well as elevating patient survival quality (Rayego-Mateos et al., 2023).

## Current therapeutic strategies for renal disease

Chronic kidney disease (CKD) is defined as clinical renal insufficiency or renal impairment, or abnormal urinary sediment, hematuria, proteinuria, or structural abnormalities of the kidney on imaging that persist for more than 3 months (August, 2023). Factors such as population aging, hypertension, diabetes, hyperuricemia, novel coronavirus infection, and cardiovascular disease are further intensifying the global disease burden of CKD (Belo and Carvalho, 2023). Currently, there is no specific drug for the treatment of chronic kidney disease. The main emphasis is to decelerate the progression of renal disease and hinder any further impairment of renal function, with the ultimate goal of preventing the occurrence of cardiovascular and renal complications in patients. With the in-depth study of kidney pathophysiology and the exploration of therapeutic agents, CKD patients now have more treatment options (August, 2023; Brody, 2023). The overactivation of the renin-angiotensin system (RASS) constitutes a crucial mechanism underlying the pathogenesis of CKD, AKI and structural impairment (Laffer et al., 2020). Inhibition of the RASS through agents such as renin inhibitors, angiotensin-converting enzyme inhibitors (ACEIs), and Ang II receptor blockers (ARBs) effectively reduces systemic blood pressure, stabilizes renal glomerular hemodynamics, mitigates mesangial stretching, podocyte injury, and glomerulosclerosis, and prevents the deteriorative effects of Ang II on both tubular and glomerular structural levels (Leon and Tangri, 2019). In the case of diabetic nephropathy, sodium-glucose co-transporter 2(SGLT-2) inhibitors (i.e., dapagliflozin) are most frequently mentioned for maintaining kidney function and preventing the progression of diabetic nephropathy to end-stage renal disease (Prattichizzo et al., 2021). Additionally, endothelin blockers, nonsteroidal mineralocorticoid receptor antagonist and glucagon-like peptide 1 receptor agonists are also very prospective and promising (Kohan and Pollock, 2013; Prattichizzo et al., 2021; González-Juanatey et al., 2023). Mesenchymal stem cells is considered one of the cutting-edge approaches to treating renal diseases (Yun and Lee, 2019). There is a host of complement inhibitors on the market and in clinical studies, covering several key aspects of the complement activation cascade, thus inhibition of complement may now offer a new means of management for an array of CKD (Kelleher and Kocinsky, 2020). Scientists have devised a strategy for the prevention and treatment of renal disease based on the regulation of intestinal flora by investigating the regulatory mechanisms of the intestinal micro-environment on renal disease (Hobby et al., 2019; Kovvuru and Velez, 2021). When entering end-stage renal disease, renal replacement therapy is the only option to extend life for patients with uremia, like hemodialysis, peritoneal dialysis, and kidney transplantation. Kidney transplantation is currently believed to be the best type of renal substitution therapy. However, the kidney donor source is tight, and there are strict matching requirements, high cost, accompanied by a myriad of complications (Kovvuru and Velez, 2021). Although treatment strategies are diverse, renal disease progression is imminent and the current treatment of CKD is limited, thus requiring the development of highly effective, multi-target agents with small side effect.

## Therapeutic effects and mechanisms of salidroside in renal diseases

### Diabetic nephropathy

Diabetic nephropathy (DN) is an important type of CKD and the prime cause of end-stage renal failure worldwide (Samsu, 2021). DN is the main cause of death in patients with type 1 diabetes, and in type 2 diabetes the serious detriment caused by DN is second only to atherosclerotic diseases of cardiovascular and cerebrovascular arteries. So early detection and early intervention is completely advantageous. Current studies have shown that the pathophysiological pathogenesis of DN is closely correlated to a variety of aspects, such as the formation of glycosylation end products caused by glucose metabolism disorder, activation of polyol pathway, lipid metabolism disorder, hemodynamic changes, inflammatory response, oxidative stress, endoplasmic reticulum stress and genetic factors (Hu et al., 2023).

It is generally accepted that hyperglycemia is the fundamental criminal to induce DN (DeFronzo et al., 2021). As previously mentioned, high blood sugar levels not only stimulate the production of ROS through AGEs, but also aggravate oxidative stress by disrupting lipid metabolism, causing DNA and protein damage in podocytes, mesangial cells, and endothelial cells (Giacco and Brownlee, 2010; Mitrofanova et al., 2023). Primarily, the effect of salidroside on reducing blood glucose and regulating blood lipid can prevent the occurrence and development of DN from the source (Li et al., 2011). It has been observed in db/db mice treated with salidroside to reduce blood glucose levels, inhibit liver gluconeogenesis by activating AMPK, down-regulating phosphoenolpyruvate carboxylase and gluconeogenic enzyme G6Pase (Wang et al., 2016). By activating the AMPK/PI3K/Akt/GSK3β signaling pathway, salidroside increases GLUT4 expression in skeletal muscle and improves insulin resistance to reduce the blood sugar (Wu et al., 2015). Heme oxygenase-1(HO-1), an inducible heme oxygenase, which is a key member of the antioxidant and anti-apoptotic family, functions in hindering inflammatory processes and oxidative tissue damage (Lever et al., 2016). High concentrations of glucose can accelerate apoptosis by inducing caspase family members, such as caspase-3 and caspase-9, and hence activation of caspase-3 and caspase-9 serves as an indicator of pro-apoptosis *in vitro* (Susztak et al., 2006). In an *in vitro* experimental study, culturing podocytes in a high glucose environment increased the apoptosis rate and promoted the expression of caspase-3 and caspase-9 and other related proteins. After treating murine podocytes with 50 μ mol/L salidroside, the expression levels of caspase-3 and caspase-9 were reduced, and salidroside were able to elevate HO-1 expression, reduce hyperglycemia-induced ROS and apoptosis, improving mouse podocytes viability (Lu et al., 2017). Podocytes are epithelial cells in the dirty layer of the renal capsule that attach to the outer glomerular basement membrane and, together with vascular endothelial cells and the glomerular basement membrane, constitute the glomerular filtration barrier function (Garg, 2018). Upon the relentless influence of hyperglycemia, podocytes are subject to a series of detrimental changes including hypertrophy, apoptosis, epithelial-to-mesenchymal transition (EMT), and autophagy (Li et al., 2023). These transformations are manifested by cytoskeletal remodeling, decline in slit diaphragms, depletion of foot-processes, and a reduction in their own number (Thomas et al., 2015; Garg, 2018). Due to the non-regenerative nature of podocytes, elucidating the mechanisms underlying podocyte injury and developing strategies for preserving their integrity have emerged as crucial research directions in kidney disease (Barutta et al., 2022). Furthermore, Important signaling pathways targeting HO-1 induction including ILK/Akt, JNK, ERK1/2, p38MAPK and Nrf2, are involved in the protective effect of salidroside on HG-cultured podocytes (Lu et al., 2017; Barutta et al., 2022).

Diabetes mellitus can induce adaptive pathological changes in the renal glomerulus, such as thickening of the glomerular basement membrane, hypertrophy and proliferation of mesangial cells, and deposition of mesangial matrix, all of which result in impaired glomerular filtration function (Lei et al., 2019). YIN et al. cultured human glomerular mesangial cells in a high glucose environment and displayed that the control group produced substantial amounts of ROS and TGF-β, and ERK1 and ERK2 were phosphorylated in glomerular mesangial cells, whereas the production of ROS and TGF-β was inhibited after incubation with salidroside, and no effect was seen on the expression of ERK1/2 (Yin et al., 2009). The activation of the ERK1/2 signaling pathway has been demonstrated to enhance mesangial cell proliferation and induce the activation of pro-inflammatory factors, such as TGF-β (Isono et al., 2000). Obviously, salidroside alleviates oxidative stress and hyperglycemy-induced mesangial cell proliferation by inhibiting multiple targets of ROS, TGF-β and ERK1/2 phosphorylation. TXNIP acts as a bridging protein that causes dysfunction and pyroptosis of pancreatic β-cells under the toxic effects of high glucose and high lipids, possessing the capacity to activate the NLRP3 inflammasome (Wondafrash et al., 2020). The decreased activity of the antioxidant system, loss of antioxidants, and excessive generation of peroxides further impair the function of pancreatic β-cells, promoting insulin resistance and accelerating the development of DN (Gerber and Rutter, 2017). The NOD-like receptor protein 3(NLRP3) inflammasome is a trimeric complex, serving as a novel regulatory factor in inflammation and pyroptosis. After induction by high concentrations of glucose, NLRP3 inflammasome can induce caspase-1 self-cleavage and enzymatic activity, thereby facilitating the activation of IL-1β and subsequently triggering inflammatory responses in the cell itself or neighboring cells (Wu et al., 2018; Hong et al., 2019). Investigation has revealed that the expression of renal AMPK is downregulated in animal models of DN, and the activity of AMPK in mesangial cells is decreased in DN patients. Pharmacological activation of AMPK has been found to improve the extent of cellular damage, lipid distribution, and insulin sensitivity, thus exhibiting a favorable protective effect on the kidneys (Ren et al., 2020). Shati conducted *in vivo* and *in vitro* experiments in STZ-induced T1DM rats and in HG-treated mesangial cell (Shati, 2020). Salidroside were demonstrated to inhibit oxidative stress and apoptosis in renal tissues in T1DM induced by STZ by the mechanism that it reduces fasting blood glucose levels, directly scavenges ROS, stimulates the activation of AMPK, and subsequently stimulates SIRT1 signaling, which leads to deacetylation of FOXO1 and P53, and ultimately the activation of Bax, and the downregulation of Bcl-2 and MnSOD. Akt is a serine/threonine-specific protein kinase with a major role in glucose metabolism and apoptosis. Phosphorylation of Akt reduces glycogen synthesis by restraining the activity of glycogen synthase kinase 3β(GSK-3β) and augments the body’s blood glucose level (Juhaszova et al., 2009). GSK-3β is involved in the regulation of apoptosis, promotes glucose metabolism and regulates cell cycle by influencing glucose levels in blood. Pei et al. reported that Salidroside (50 mg/kg/day, oral) was given to STZ-induced DN rats for 8 weeks, which could significantly counteracted the glucose-raising effect of STZ and reduce the levels of blood creatinine, urea nitrogen and urinary protein in DN rats (Pei et al., 2022). At the same time, the pathological changes of kidney can be alleviated by improving the abnormal glomerular tissue, swelling and mesangial matrix dilation. Salidroside has the potential to activate the Akt/GSK-3β signaling pathway in the kidney of rats with DN, indicating that increased levels of phosphorylated Akt and GSK-3β can suppress the production of inflammatory factors, mitigate oxidative stress and inflammatory response, enhance cellular glucose uptake, and alleviate apoptosis, thereby ameliorating renal tissue injury.

Mitochondria serve as the primary site for cellular aerobic respiration, providing a substantial amount of energy to the organism (Ho and Shirakawa, 2022). Mitochondrial fusion defects, excessive division, impaired autophagy or excessive autophagy, biosynthesis dysfunction, and oxidative stress can lead to alterations in mitochondrial quality, quantity, and structure (Ahmad et al., 2021; Tang et al., 2021). Consequently, mitochondrial dysfunction ensues, resulting in inadequate renal energy supply and oxidative stress-induced damage that disrupts normal kidney structure and function (Bhargava and Schnellmann, 2017). These factors are closely associated with the onset and progression of DN. Targeting mitochondria with various drugs has emerged as a promising approach to improve renal energy metabolism and mitigate renal pathological changes, thereby delaying DN progression (Rahman et al., 2022). In a study by Haiyan Xue et al., obese mice with STZ-induced kidney injury were treated with salidroside (supplemented in drinking water at concentrations of 0.3 mg/mL or 0.6 mg/mL) for 10 weeks. This treatment led to reduced urinary albumin levels along with decreased BUN and Scr levels in the model mice. Salidroside also reversed STZ-induced reduction of nephrin and podocin expression while alleviating podocyte injury and renal fibrosis through activation of the Sirt1/PGC-1α signaling pathway, thus promoting mitochondrial biogenesis and exerting protective effects against DN (Xue et al., 2019). Studies have suggested that proximal tubular lesions may occur earlier than glomerular lesions in DN, which in turn leads to irreversible glomerular damage. The nephroprotective effect of salidroside is partially attributed to its ability to suppress apoptosis in proximal tubular cells by targeting the apoptotic protein BIM during this process. Notably, salidroside significantly attenuated HG-induced upregulation of BIM mRNA expression along with BAX activation and cleaved caspase-3 protein and mitigated apoptosis of proximal tubular epithelial cells (Guo et al., 2018). The underlying mechanisms by which salidroside inhibits BIM mRNA expression in DN are incompletely understood, Guo et al. additionally analyzed BIM-related targets through protein-protein networks and pathways, suggesting that JNK, ERK and PI3K/AKT signaling pathway may be involved in regulating BIM transcriptional expression.

HG can lead to apoptosis of glomerular endothelial cells (GECs) and mitochondrial damage, and renal hypoxia is an indispensable pathological mechanism of DN (Catrina and Zheng, 2021). Cellular adaptation to hypoxia is mediated by the transcription factors HIF-1α and HIF-2α. Both HIF-1α and HIF-2α upregulate the expression of multiple glycolytis-related genes to increase aerobic glycolysis (Ramakrishnan and Shah, 2017; Yao et al., 2021). Pharmacological activation targeting the HIF system has been demonstrated in diabetic patients and animal models as a potential clinical target for the prevention and treatment of diabetes (Nordquist et al., 2015; Catrina and Zheng, 2021). One study found that salidroside combined with FG-4592 (prolyl hydroxylase inhibitor) increased cell viability and decreased apoptosis rate by decreasing the expression of PHD-1, upregulating HIF-1α, HIF-2α and downstream gene VEGFA in high-glycemia-induced GECs injury (Xie et al, 2019). Proteinuria is an important sign of kidney damage, and the amount of urinary protein is tightly associated with the degree of diabetic kidney damage and disease progression (de Zeeuw, 2007). Currently, the mechanism of proteinuria production has been less studied on GECs and mainly on the glycocalyx that covers the surface of endothelial cells. Some reports have shown that high glucose leads to increased permeability of GECs to albumin by impairing the glycocalyx (Singh et al., 2011). The Caveolae, a depression-like lipid raft structure formed by cytoplasmic invagination on endothelial cells, resembles a “flask,” and its signature protein is the Caveolin-1(Cav-1) (Luo et al., 2021). The transport of albumin in microvascular endothelial cells is mainly mediated by caveolae, and caveolin-1/caveolae is also expressed in GECs (Minshall et al., 2002; Moriyama et al., 2011). A study showed that hyperglycemia promotes albumin penetrance by upregulating the ROS/Src/Cav-1 pathway and downregulating the AMPK/Cav-1 pathway. Salidroside block hyperglycemia-induced albumin cell-penetration by a mechanism achieved through the dual action of antioxidant effects and activation of AMPK, thus exerting a palliative effect on proteinuria (Wu et al., 2016). Overall, the administration of salidroside not only demonstrates an externally marked hypoglycemic and hypolipidemic effect but also intrinsically ameliorates DN by activating the AMPK/SIRT1, Nrf2/HO-1, Akt/ILK, Sirt1/PGC-1α, and Akt/GSK-3β, AMPK/Cav-1 pathways while suppressing the TXNIP-NLRP3, ERK1/2, and TGF-β1/Smad, ROS/Src/Cav-1 pathways to suppress cellular apoptosis, oxidative stress, and inflammation.

### Renal ischemia-reperfusion injury

Renal ischemia reperfusion injury (RIRI) refers to a pathological phenomenon in which renal tissue suffers from ischemia for a certain period of time, and after restoration of blood supply, the degree of tissue damage aggravates instead (Weight et al., 1996). RIRI often occurs during great vessels surgery, shock, organ transplantation, complicated renal stone extraction, extracorporeal shock wave lithotripsy and renal tumor preservation surgery, which may lead to AKI (Zhao et al., 2018). Consequently, RIRI is one of the determinants that alter patient survival and graft survival, and how to effectively averted RIRI and foster the recovery of renal function has become an compelling research direction (Zhao et al., 2018). The kidney has 1/4–1/5 of the blood flow of the heart, is a hyperperfused organ, which is very sensitive to ischemia-reperfusion (Edwards and Kurtcuoglu, 2022). After prolonged ischemia, reperfusion not only fails to revive tissue structure and function, but also exacerbates renal insufficiency and structural defects. For the time, the pathogenesis of ischemia-reperfusion has not yet been thoroughly elucidated, but considerable research data show that it may be due to excessive production or unfavorable removal of oxygen radicals, which can lead to membrane lipid peroxidation, intracellular calcium overload, energy depletion, cytokine and leukocyte adhesion, and cytoskeletal disorders (Tsuda et al., 2012; Rovcanin et al., 2016; Sakai et al., 2019).

In recent years, with the rapid improvement of the understanding of cytokine network, the role of cytokines in ischemia-reperfusion of related organs has been paid more and more attention. Ischemia reperfusion exacerbates kidney injury through inflammatory response, which is mainly mediated by cytokines such as IL-1, IL-6, TNF-α, chemokines and leukocyte infiltration (Donnahoo et al., 1999; Patel et al., 2005; Prieto-Moure et al., 2017). It has been reported that the NF-κB signaling pathway in RIRI is abnormally activated, which leads to the production of inflammatory factors (Xiao et al., 2020). An *in vitro* study found that human renal tubular epithelial cells (HK-2) under I/R conditions, given Sal intervention, showed improvement in certain oxidative stress indicators such as SOD, MDA, and related cytokines such as IL-1β, IL-6, and TNF-α. It also inhibited apoptosis in HK-2 cells by increasing Bcl-2 expression while decreasing Bax expression and caspase-3 activity. Salidroside inhibited hypoxia/reoxygenation-induced oxidative stress, inflammatory response and apoptosis in human renal tubular epithelial cells by regulating the TLR4/NF-kB signaling pathway, suggesting that salidroside may have beneficial effects on renal ischemia-reperfusion injury (Sun et al., 2018). A renal ischemia-reperfusion model was established by ligating bilateral renal arteries in rats for 45 min, followed by the removal of hemostatic forceps to restore blood flow. Preoperative intragastric administration of salidroside (10 mg/kg and 100 mg/kg) effectively mitigated kidney injury through the inhibition of oxidative stress and ferroptosis (Tang et al., 2023). *In vitro* cell experiments demonstrated that this protective effect was mediated via activation of the PI3K/AKT signaling pathway in renal tubular epithelial cells.

The kidney serves not only as a crucial excretory organ, but also possesses an endocrine function by secreting essential hormones such as erythropoietin (EPO), which is of paramount importance (Copur et al., 2023). The majority of EPO in the human body is secreted by peritubular interstitial cells located in the renal cortex (Kishore et al., 2007). EPO can promote the production of red blood cells and thus increase the oxygen-carrying capacity of blood. When the body is in a state of ischemia and hypoxia, renal EPO secretion can be enhanced. Hypoxia-inducible factor1α(HIF-1α) is a sensitive factor to ischemia and hypoxia, composed of two subunits, α and β (Ke and Costa, 2006). HIF-1α acts as an oxygen regulator that is strictly regulated by partial pressure of oxygen (Masoud and Li, 2015). Under normal oxygen conditions, proline residues on HIF-1α are hydroxylated by proline hydroxylase before being degraded through protein ubiquitination hydrolysis system after binding with VHL protein. However, under hypoxic conditions, inhibition of HIF-1α hydroxylation leads to increased levels of cytoplasmic HIF-1α and enhanced transcriptional activity. This complex binds with HIF-1β to form the HIF-1 complex that activates downstream genes such as vascular endothelial growth factor (VEGF) via binding with hypoxia reaction elements in the nucleus. As a result, this triggers a series of adaptive reactions in tissue cells resistant to low-oxygen environments (Cernaro et al., 2019). As HIF-1α regulates transcriptional activity for EPO gene expression, evaluating expression degree for HIF-1α can determine EPO expression levels. Zheng and co-workers cultivated renal fibroblasts (HEK293T) with aqueous extracts of roots and rhizomes of R. crenulate collected from Tibet, China, and its extracts blocked the renal fibroblast degradation pathway inducing the accumulation of HIF-1α, which induced the expression of EPO at both the mRNA and protein levels (Zheng et al., 2011). In addition, the induction of EPO by Rhodiola was also observed in cultured hepatocytes, since the liver is another important organ besides the kidney that provides EPO regulation. They later extended their hypothesis that salidroside in Rhodiola, may be the main chemical responsible for producing this anti-hypoxic effect. It was found that salidroside-induced EPO expression was mediated by HIF-1α, but not HIF-2α, which activates the transcription of the EPO gene through HRE regulatory elements. Salidroside accumulate HIF-1α protein by blocking the protein degradation pathway may be at the stage of blocking the hydroxylation of HIF-1α, but fail to affect the transcriptional level of HIF-1α (Zheng et al., 2012).

RIRI, as an acute tissue injury, can stimulate and activate nonspecific immune cells, resulting in an immune stress response (Nakamura et al., 2019). Therefore, positively controlling the inflammatory response and maintaining immune homeostasis is one of the effective ways to alleviate RIRI. Previous studies have demonstrated that salidroside can increase the secretion of Th1- and Th2-type cytokines in mouse serum, and additionally attenuate ulcerative colitis and cerebral ischemia-reperfusion injury by regulating Th17/Treg imbalance (Lin et al., 2011; Yin et al., 2021; Liu et al., 2023). Based on these results, we speculate that salidroside may have a positive effect on immunotherapy of renal ischemia-perfusion, but there are no more experiments to verify this. The effect of salidroside in the treatment of RIRI is in the limelight. It has been suggested that it can attenuate ischemic perfusion injury in several organs, such as brain tissue, heart, liver, kidney, etc., and promote the recovery of organ physiological functions (Cai et al., 2017; Han et al., 2022). Based on the outstanding anti-hypoxia and anti-fatigue ability of salidroside, we believe that we can explore more animal models such as renal transplantation ischemia model as well as exercise renal failure to explore the therapeutic potential of salidroside in RIRI from multiple perspectives and mechanisms, and to expand the application of salidroside in military medicine, sports medicine, and clinical medicine.

### Renal fibrosis

Renal fibrosis (RF) is the common pathway of CKD to end-stage renal failure caused by various causes, mainly manifested as glomerulosclerosis, renal tubule interstitial fibrosis (Nogueira et al., 2017; Nastase et al., 2018). The prime pathological changes of renal fibrosis are ECM deposition, inflammatory cell infiltration, progressive nephron reduction (including podocyte apoptosis, tubular epithelial cell shedding, tubular atrophy), leading to the destruction of normal nephron structure and loss of renal function (Nogueira et al., 2017). Although the molecular mechanism of renal fibrosis has not been clearly studied, tubular epithelial cell apoptosis, ECM deposition, tubular EMT, cell senescence and dedifferentiation are all very important processes in the slow progression from CKD to fibrosis (Lovisa et al., 2015; Rayego-Mateos et al., 2021; Shu et al., 2021).

Renal tubular epithelial cells with abundant mitochondria are high oxygen-consuming cells which are more vulnerable to oxidative stress than other renal cells (Liu et al., 2022). When mitochondria are damaged, their membrane potential drops and the permeability of the inner membrane increases, leading to disruption of energy metabolism and further causing cell death. The persistent unremitting inflammatory response after kidney injury initiates the fibrotic process, promoting the activation of renal tubular interstitial cells, followed by the activation of multiple fibrotic factors and vasoactive substances (Chung et al., 2019). This gives rise to ECM accumulation, tubular atrophy, microvascular degeneration, chronic hypoxia of renal tissue, and the replacement of normal renal tissue by scar tissue conclusively developing into renal failure (Huang et al., 2023). Studies have confirmed that oxidative stress is a major factor in renal fibrosis during unilateral ureteral obstruction (UUO) (Aranda-Rivera et al., 2021). UUO leads to hypoxia and thus oxidative stress, and mitochondrial dysfunction caused by oxidative stress plays a key role in the development of UUO fibrosis, suggesting that mitochondria may play an active role in the process of renal fibrosis in CKD and are the earliest targets to be involved, leading to the speculation that drugs that protect mitochondrial function may have a role in delaying renal fibrosis. It has already been mentioned that salidroside can improve mitochondrial biogenesis and reduce renal fibrosis in diabetic patients by activating the Sirt1/PGC-1α signaling pathway (Xue et al., 2019). In addition, Ugur Uyeturk demonstrated the alleviating effect of rhodiola extract on renal injury due to UUO in rats, but the exact mechanism of the effect deserves further study (Uyeturk et al., 2013).

Salidroside can regulate the progression of renal fibrosis by interfering with the expression of various inflammatory cytokines and related signaling pathways. It was found that all types of kidney cells secreted TGF-β1 and expressed TGF-β receptors (Hu et al., 2018). TGF-β is an important fibroblast factor, which is one of the important reasons for the transdifferentiation of renal tubular epithelial cells into mesenchymal cells (Iwano, 2010). TGF-β1 is often highly expressed in renal fibrosis, which promotes the occurrence and progression of renal failure by promoting downstream Smad2 and Smad3 and inhibiting the expression of Smad6 and Smad7 (Nelson et al., 2011). Many studies have confirmed that salidroside can inhibit its expression to improve fibrosis and delay disease progression (Tang et al., 2016; Feng et al., 2018; Ma J. et al., 2023). Qi et al. found that salidroside could reduce the contents of blood creatinine, urea nitrogen and urinary albumin in DN rats, increase the activities of enzymes related to oxidative defense system such as SOD, CAT and GPX, and significantly reduce the expression levels of TNF-α, IL-1β and IL-6. At the same time, the expression levels of fibrosis-related proteins such as TGF-β1, p-Smad2, and p-Smad3 in the kidney decreased significantly, suggesting that salidrosdie slows down the pathological changes of kidney tissue and improves renal sclerosis and fibrosis in DN rats by inhibiting the TGF-β1/Smad2/3 pathway (Qi et al., 2021). EMT refers to the biological process by which epithelial cells are transformed into interstitial cells through a specific procedure (Iwano, 2010). Under the stimulation of inflammatory factors and pro-fibrotic factors, cells lose their inherent phenotype and gradually transform into the interstitial, which is of great significance for the occurrence and development of fibrosis (Lovisa et al., 2015). After being activated by TLR4, NF-κB translocates to the nucleus to induce the expression of specific genes, triggering the massive release of inflammatory factors and causing renal fibrosis (Liu et al., 2018). Li et al. established UUO or folic acid-induced mouse renal interstitial fibrosis *in vivo*, and established TGF-β1-stimulated human proximal renal tubular epithelial cell (HK-2) model *in vitro*. After salidroside treatment, the expressions of type III collagen, type I collagen, α-SMA, TGF-β1 and vimentin in mouse kidney tissue were significantly decreased, and the expression of E-cad was increased, which significantly inhibited EMT (Li R. et al., 2019). Mechanically, salidroside can reduce the protein levels of EMT markers in mouse kidney and HK-2 cells, and significantly reduce the release of inflammatory factors, which is related to the inhibition of TLR4/NF-κB and MAPK signaling pathways.

The involvement of the Wnt/β-catenin signaling pathway in the progression of renal fibrosis is becoming increasingly evident. Activation and engagement of the Wnt/β-catenin signaling pathway have been observed in apoptosis and EMT of mesangial cells, dysfunction of podocytes, as well as EMT of renal tubular cells, subsequently leading to renal fibrosis and interstitial fibrosis (Zhou et al., 2013). There is cross-regulation between Wnt and TGF-β signaling pathway. TGF-β signaling pathway activates Wnt/β-catenin signaling pathway through the production of Wnt protein, while activated Wnt/β-catenin stabilizes TGF-β1/Smad signaling pathway. The co-activation of the two pathways can effectively elicit the fibrosis response (Działo et al., 2021). Huang et al. used the mouse model of adriamycin (ADR) nephropathy to explore the pharmacological mechanism of salidroside. Salidroside reversed the massive accumulation of β-catenin in the nucleus of ADR nephropathy, decreased the expression of nephrin and podocin, and improved proteinuria, renal fibrosis and podocyte injury in ADR nephropathy (Huang et al., 2019). Shati et al. also demonstrated that salidroside mitigates kidney damage and fibrosis in diabetic rats, restoring normal kidney structure and function through inhibition of TGF-β1/Smad and Wnt1/3a/β-catenin signaling pathways (Shati and Alfaifi, 2020). Daily administration of salidroside (100 mg/kg) via oral gavage to STZ-induced T1DM rats significantly increased the levels of p-β-catenin, SOD, and GSH levels, while suppressing Axin-2 levels, fibronectin expression, and mRNA/protein production of collagen IIIa. Liu et al. used a mouse model of ADR nephropathy to explore the mechanism of salidroside in focal segmental glomerulosclerosis. Salidroside injection can improve proteinuria and renal function, inhibit the expression of α-SMA and fibronectin, and downregulate the expression of HIF-1α. Immunohistochemistry, Western blotting, and rt-PCR analyses collectively suggested that the PI3K/Akt/HIF-1α pathway represents a potential target for salidroside intervention, ultimately mitigating ROS production and inflammatory response, thereby effectively retarding focal segmental glomerulosclerosis (Liu and He, 2019).

The progression of renal fibrosis is closely related to aging, which eventually leads to renal dysfunction (Hommos et al., 2017). Salidroside is believed to have a wide range of anti-aging effects. In a study to evaluate the protective effect of salidroside on renal interstitial fibrosis in the context of aging and its mechanisms, the researchers selected SAMP8, a mouse with accelerated aging (Yang et al., 2022b). After 12 weeks of administration with salidroside and Ferrostatin 1, the results showed that the elevated BUN and Scr levels were significantly reduced, the serum albumin levels were increased, and mesangial hyperplasia was significantly reduced in the salidroside group. The levels of TGF-β and α-SMA in SAMP8 mice were significantly reduced. Salidroside treatment significantly reduced renal lipid peroxidation, delayed renal aging and inhibited glomerular fibrosis by regulating iron transport-related proteins and ferroptosis-related proteins. Elevated levels of ferritin can trigger autophagy, a cellular process responsible for the degradation of ferritin to enhance iron levels and induce ferroptosis (Bogdan et al., 2016; Gao et al., 2016). A recent review highlights the role of autophagy in regulating critical pathways associated with iron metabolism and lipid peroxidation, amplifying the positive feedback loop of ferroptosis (Lee et al., 2023). And impaired autophagy is also a central feature of kidney aging, which led us to ponder whether salidroside can also delay renal aging or renal fibrosis through by regulating autophagy, necessitating further experimental validation. Taken together, these experiments provide compelling evidence supporting the potential therapeutic efficacy of salidroside administration in the treatment of renal fibrosis by modulating multiple pathways including Sirt1/PGC-1α, PI3K/Akt/HIF-1α, Wnt1/Wnt3a β-catenin, TGF-β1/Smad2/3, TLR4/NF-κB, MAPK signaling pathways and ferroptosis.

### Renal cell carcinoma

Renal cell carcinoma (RCC) is a common kidney cancer originating from tubular epithelial cells of the kidney, and it is also one of the most predominant malignant tumors of the urinary system (Perazella et al., 2018). Patients with renal cell carcinoma account for more than 95% of the total incidence of kidney cancer and approximately 3%–5% of the total incidence of cancer worldwide. Since RCC is a chemotherapy resistant cancer and surgical intervention often fails to eradicate cancer cells, targeted therapy has become the standard first-line treatment for advanced renal cancer over the past decade, but drug resistance eventually develops in almost all patients (Makhov et al., 2018; Zhang et al., 2024).

As known to all, salidroside has anticancer properties which can curb the malignant biological behaviors of many tumor cells, and induce apoptosis in multiple cancers (gastric, breast, lung, colon, bladder, glioma, etc.) (Fan et al., 2016; Li et al., 2018; Rong et al., 2020; Ma et al., 2021). Except for inhibiting the growth of cancer cells, it can likewise prohibit tumor migration, invasion, and neovascularization (Kang et al., 2018). Cai Lv et al. conducted *in vivo*/*in vitro* experiments using human renal cancer cell lines A498 and 786-O as well as the A498 xenograft tumor animal model, and concluded that salidroside could inhibit the proliferation of human renal cancer cells and induce apoptosis in a concentration- and time-dependent manner. Meanwhile, salidroside could restrain the growth of subcutaneous renal cancer tissues in nude mice and trigger apoptosis in these tissues. The main mechanism is that salidroside induced significant G1 cell cycle arrest and apoptosis in A498 and 786-0 cells, and reduced the phosphorylation level of JAK2 and STAT3, which are key proteins in the JAK2/STAT3 signaling pathway, and thus exhibited potent anticancer properties in renal cell carcinomas (Lv et al., 2016). Salidroside in patients with RCC may provide a promising treatment strategy for this malignancy. In reality, our comprehension of cancer has undergone a fundamental shift, recognizing it as not merely a disease but rather a sophisticated ecosystem (Wang Q. et al., 2023). The tumor microenvironment of renal cell carcinoma comprises not only extensive infiltrating immune cells and numerous pathologically activated tumor-related fibroblasts but also dysfunctional vascular endothelial cells and other cellular components, along with non-cellular components such as soluble factors and extracellular matrix (Lai et al., 2021). Currently, the investigation on the impact of salidroside in renal cell carcinoma is primarily focused on its effects on cell proliferation and apoptosis. However, further research is warranted to explore the influence of salidroside on additional characteristics of human renal carcinoma including invasive activity, migration ability, chemotherapy resistance, and regulation of the tumor microenvironment. Moreover, comprehensive studies are needed to elucidate the mechanism of action of salidroside by examining alterations in upstream and downstream proteins within the JAK2/STAT3 signaling pathway as well as other potential signaling pathways.

### Acute kidney injury

Acute kidney injury (AKI) is characterized by necrosis or apoptosis of renal tubular epithelial cells under the action of a series of injurious factors, leading to renal failure within a short period of time, and the most important pathological feature is acute necrosis of renal tubules (Guo et al., 2022). Critical infections, sepsis, major surgery, low cardiac output, inadequate tissue perfusion, and nephrotoxic drugs can lead to the occurrence of AKI, which is highly prevalent in ICU patients, and the incidence of AKI is 7% in patients hospitalized in general wards, whereas the incidence of AKI in critically ill patients can be as high as 36%–67% (Dennen et al., 2010). AKI that develops during sepsis represents one of the most frequent and severe complications observed throughout the course of this disease (Peerapornratana et al., 2019).

In order to investigate the protective effect of salidroside on septic acute kidney injury and its underlying mechanism, Fan et al. established a rat model of sepsis by performing cecum ligation and perforation, and injected salidroside via tail vein for 3 consecutive days in advance. The results demonstrated a significant reduction in mortality rate among the septic rats treated with salidroside, along with notable improvements in renal function and histopathological injuries (Fan et al., 2022). Septic acute kidney injury is a multifactorial condition characterized by hemodynamic changes, microcirculatory disturbances, tubular epithelial cell injury, coagulation dysfunction, resulting in reduced renal artery perfusion or hypoperfusion, tubular dysfunction, and ultimately decreased glomerular filtration rate (Basile et al., 2012). Upon exposure to endotoxins or endotoxin-like substances released by bacteria, white blood cells as well as renal tubule skin cells and epithelial cells initiate complex immune responses that involve the release of various inflammatory mediators into circulation (Poston and Koyner, 2019). Previous studies have emphasized the significant anti-inflammatory effects of salidroside in sepsis, notably, this study emphasizes its regulatory role within septic AKI’s complex immune network inflammation by reducing plasma and kidney levels of TNF-α, IL-1β, and IL-17A, which are pivotal cytokines involved in the pathogenesis of sepsis (Liu et al., 2015; Lan et al., 2017). Inflammatory conditions lead to kidney injury characterized by increased expression levels of Scr, BUN, KIM-1, and neutrophil gelatinase-associated lipocalin protein. These alterations can be significantly attenuated by salidroside treatment within the kidneys through inhibition of inflammation and modulation apoptosis levels within renal tubular epithelial cells via downregulation NF-kB pathway regulation apoptosis-related gene expression. Afterwards, Pan et al. administered intraperitoneal injections of salidroside to LPS-induced AKI mice only 2 h prior and demonstrated potent anti-inflammatory effects, as well as improvements in renal failure indices such as BUN, Scr, NGAL, and KIM-1 (Pan et al., 2023). In addition, *ex vivo* experiments demonstrated that salidroside attenuated LPS-induced apoptosis in renal tissues and podocytes, inhibited oxidative stress in renal tissues and enhanced autophagy in podocytes. Among them, the activation of the SIRT1/Nrf2 pathway played a pivotal role in mediating these alterations. Numerous studies have substantiated that activating SIRT1 can mitigate the inflammatory response induced by sepsis and confer protection against acute kidney injury (Wei et al., 2019; Deng et al., 2021). SIRT1, an NAD + dependent histone deacetylase, is considered to be one of the major regulatory molecules of sepsis induced acute kidney injury due to its role in reducing oxidative stress and inflammation (Wei et al., 2019). Moreover, SIRT1 may upregulate Nrf2 expression to diminish ROS production (Zhang et al., 2021). The downstream target genes regulated by Nrf2 signaling pathway primarily encompass SOD, GPX, CAT, HO-1, which play a crucial role in the body’s resistance to oxidative stress (Nezu and Suzuki, 2020).

### Other kidney injury

Xing Y et al., discovered that salidroside prevented contrast agent-induced renal injury in rats through an anti-oxidative stress mechanism involving increased gene and protein expression of SOD and endothelial-type nitric oxide synthase along with elevated nitric oxide synthase activity and nitric oxide levels while decreasing MDA, Ang II levels as well as 8-hydroxy-2′-deoxyuridine levels (Xing et al., 2015). Lei et al. found that at concentrations of 10, 50 and 100 μmol/L, salidroside could reverse the hypertrophy and elongation of proximal renal tubular epithelial cells induced by cobalt chloride in hypoxic rats, showing the appearance of myoblasts. Salidroside inhibits the expression of TGF-β1 and HIF-1α, reduces the increase of extracellular matrix such as α-SMA, collagen I and fibronectin, and inhibits the transdifferentiation of hypoxic renal tubular epithelial cells-myofibroblasts (Zhang et al., 2010).

## Conclusion and future perspective

In recent years, many experts and scholars have studied the renal disease mechanism of salidroside, but no detailed systematic summary has been found. Here, the pharmacological action and mechanism of salidroside in the kidney in recent years are reviewed to provide reference for the research and development of new drugs and clinical drugs based on salidroside, and bring fresh insight for the treatment of renal diseases. As discussed above, salidroside demonstrated good renal protection against hyperglycemia, ischemia reperfusion, cobalt chloride, streptozotocin, ureteral obstruction, and kidney injury caused by contrast agent through antioxidant and anti-inflammatory effects. However, the current research on the pharmacological effects of salidroside mainly focuses on *in vivo* and *in vitro* experiments, and clinical studies are extremely limited.

**FIGURE 3:**
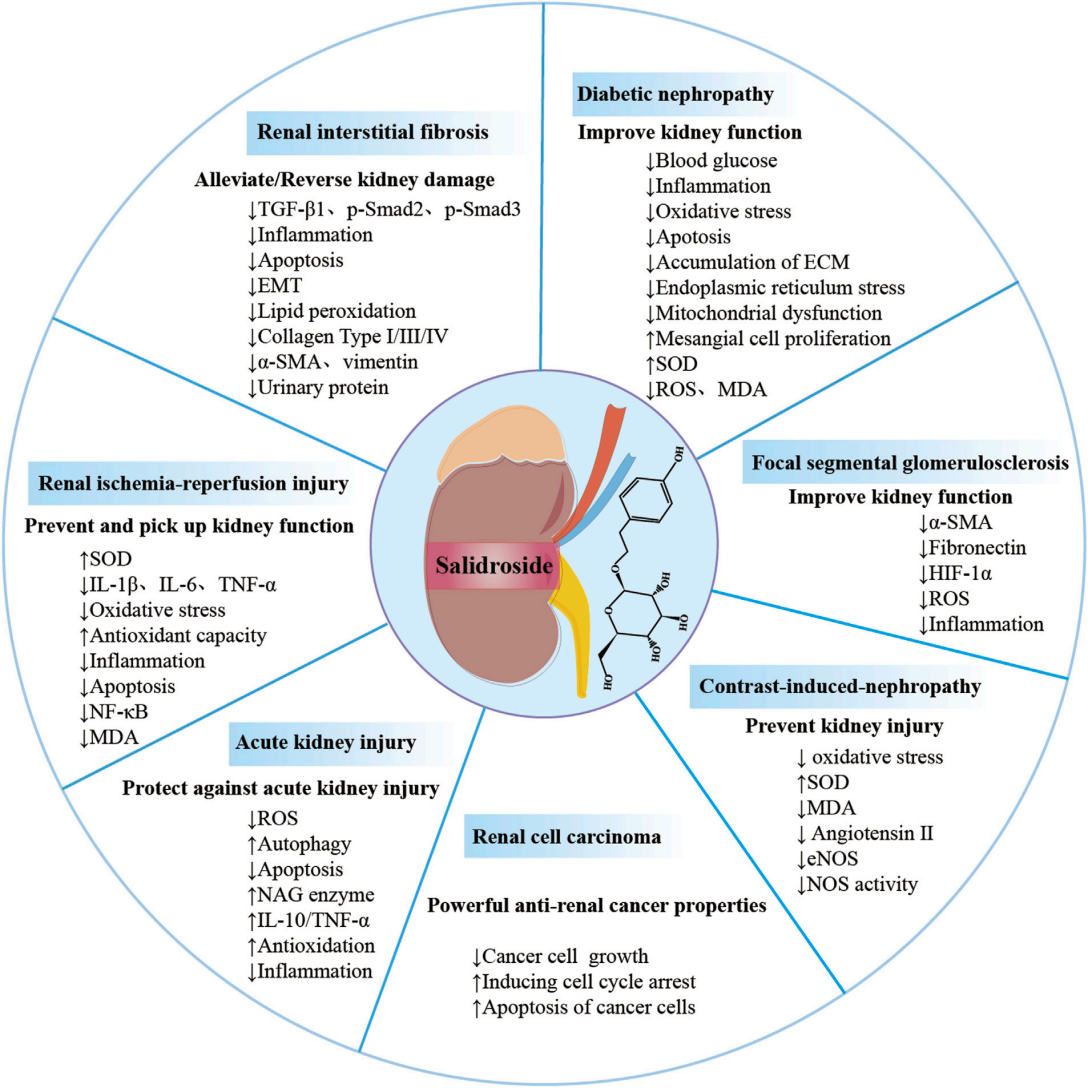
Overview of salidroside in renal diseases. Salidroside can prevent and treat kidney damage in various renal diseases, such as acute kidney injury, diabetic nephropathy and renal ischemia-perfusion injury, renal interstitial fibrosis, renal cell carcinoma, focal segmental glomerulosclerosis, contrast-induced nephropathy.

**FIGURE 4:**
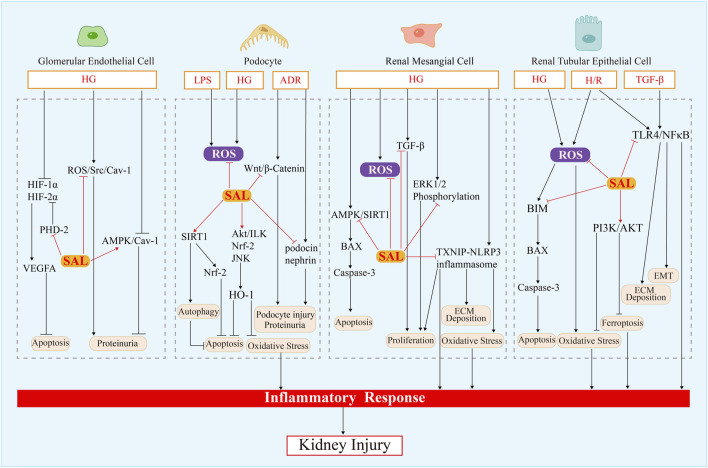
Mechanism of salidroside action in renal intrinsic cells.

For the most part, salidroside has a potential protective effect on renal function and pathological impairment, involving apoptosis, autophagy, regulation of the kidney micro-inflammation, anti-oxidative stress and anticancerous, and the therapeutic effect is encouraging, to say the least. Specifically, the potential mechanisms are primarily associated with the regulation of gene and protein expression in glomerular endothelial cells, podocytes, renal tubule cells, renal mesangial cells and renal cell carcinoma cell, including TNF-α, TGF-β, IL-1β, IL-17A, IL-6, MCP-1, Bcl-2, VEGF, ECM protein, caspase-3, HIF-1α, BIM, as well as the modulation of AMPK/SIRT1, Nrf2/HO-1, Sirt1/PGC-1α, ROS/Src/Cav-1, Akt/GSK-3β, TXNIP-NLRP3, ERK1/2, TGF-β1/Smad2/3, PI3K/Akt, Wnt1/Wnt3a β-catenin, TLR4/NF-κB, MAPK, JAK2/STAT3, SIRT1/Nrf2 pathways (Figures 3, 4). Indeed, the specific signaling pathways implicated in salidroside’s anti-inflammatory, anti-fibrotic, and antioxidant effects necessitate comprehensive investigations into the precise molecular mechanisms underlying its therapeutic potential for renal injury at both cellular and animal levels. Given the rich pharmacological effects of salidroside, there is a compelling rationale to explore its mechanisms of action in regulating key factors such as endoplasmic reticulum stress, mitochondrial dysfunction, autophagy, immune senescence, and other processes associated with kidney diseases. Furthermore, we also can explore whether salidroside participates in the renal tumor microenvironment through regulating biological behaviors (invasiveness, migratory ability), chemotherapy resistance, metabolism, and immunity. Animal and cell experiments utilizing models of exercise-induced kidney injury, drug-induced kidney injury, kidney transplantation injury, infectious kidney injury, and kidney aging can greatly contribute to our understanding of the potential applications of salidroside in the management of kidney diseases. There are fewer studies on salidroside preparations and the combination of salidroside with other drugs, and the efficacy and safety need to be further investigated. Therefore, there is still a broad research and development space for the development and utilization of salidroside in renal diseases.

## Figures and Tables

**Table:** 

**ADR**	Adriamycin
**AKI**	Acute kidney injury
**Ang II**	angiotensin II
**BUN**	blood urea nitrogen
**CKD**	chronic kidney disease
**DN**	Diabetic nephropathy
**ECM**	extracellular matrix
**E-cad**	E-cadherin
**EMT**	epithelial-mesenchymal transformation
**EPO**	erythropoietin
**GECs**	glomerular endothelial cells
**HG**	high glucose
**HIF-1α**	hypoxia-induciblefactor-1α
**HK-2**	Human renal tubular epithelial cells
**HO-1**	Hemeoxygenase-1
**H/R**	hypoxia/reoxygenation
**I/R**	ischemia/reperfusion
**LPS**	lipopolysaccharide
**MDA**	malondialdehyde
**NLRP3**	NOD-like receptor protein3
**Nrf2**	nuclearfactorerythroid2-relatedfactor2
**PTECs**	proximal tubular epithelial cells
**RCC**	renal cell carcinoma
**RIRI**	renal ischemia reperfusion injury
**SAL**	salidroside
**Scr**	serum creatinine
**SOD**	Superoxide dismutase
**STZ**	streptozotocin
**TC**	total cholesterol
**TGF-β**	transforming growth factor-β1
**TXNIP**	thioredoxin-interacting protein
**UUO**	unilateral ureteral obstruction
**α-SMA**	α-smooth muscle actin
**NGAL**	neutrophil gelatinase-associated lipocalin
**KIM-1**	kidney injury molecule-1
